# Knowledge, Attitude and Practice about Dengue Fever among Patients Experiencing the 2017 Outbreak in Vietnam

**DOI:** 10.3390/ijerph16060976

**Published:** 2019-03-18

**Authors:** Huong Van Nguyen, Phung Quoc Tat Than, Tu Huu Nguyen, Giang Thu Vu, Chi Linh Hoang, Tung Thanh Tran, Nu Thi Truong, Son Hoang Nguyen, Huyen Phuc Do, Giang Hai Ha, Huong Lan Thi Nguyen, Anh Kim Dang, Cuong Duy Do, Tung Hoang Tran, Bach Xuan Tran, Carl A. Latkin, Cyrus S. H. Ho, Roger C. M. Ho

**Affiliations:** 1 Department of Neuroscience, Hanoi Medical University, Hanoi 100000, Vietnam; vanhuong73@hotmail.com; 2 Center of Excellence in Behavior Medicine, Nguyen Tat Thanh University, Ho Chi Minh 70000, Vietnam; selena.coentt@gmail.com (P.Q.T.T.); chi.coentt@gmail.com (C.L.H.); nu.coentt@gmail.com (N.T.T.); pcmrhcm@nus.edu.sg (R.C.M.H.); 3 Anesthesia and Critical Care Department, Hanoi Medical University, Hanoi 100000, Vietnam; nguyenhuutu@hmu.edu.vn; 4 Center of Excellence in Evidence-Based Medicine, Nguyen Tat Thanh University, Ho Chi Minh 70000, Vietnam; giang.coentt@gmail.com (G.T.V.); tung.coentt@gmail.com (T.T.T.); 5 Center of Excellence in Health Services and System Research, Nguyen Tat Thanh University, Ho Chi Minh 70000, Vietnam; son.coentt@gmail.com (S.H.N.); huyen.coentt@gmail.com (H.P.D.); 6 Institute for Global Health Innovations, Duy Tan University, Da Nang 55000, Vietnam; giang.ighi@gmail.com (G.H.H.); huong.ighi@gmail.com (H.L.T.N.), kimanh.ighi@gmail.com (A.K.D.); 7 Department of Infectious Diseases, Bach Mai Hospital, Hanoi 100000, Vietnam; doduy.cuong@gmail.com; 8 Department of Lower Limb Surgery, Vietnam-Germany Hospital, Hanoi 100000, Vietnam; tranhoangtung.vd@gmail.com; 9 Institute for Preventive Medicine and Public Health, Hanoi Medical University, Hanoi 100000, Vietnam; 10 Johns Hopkins Bloomberg School of Public Health, Baltimore, MD 21205, USA; carl.latkin@jhu.edu; 11 Department of Psychological Medicine, National University Hospital, Singapore 119074, Singapore; cyrushosh@gmail.com; 12 Department of Psychological Medicine, Yong Loo Lin School of Medicine, National University of Singapore, Singapore 119077, Singapore

**Keywords:** dengue fever, knowledge, attitude, practice, associated factors, Vietnam

## Abstract

There is a gap in the literature on the understanding of the general Vietnamese population toward dengue fever (DF). This study aimed to explore knowledge, attitudes, practice (KAP) of dengue fever among Vietnamese participants and the potential associated factors. A cross-sectional study was conducted among 330 patients at the Bach Mai Hospital in Northern Vietnam. A Tobit regression model was utilized to investigate the associated factors. The average knowledge score was 4.6/19. Respondents perceived their risk of DF infection to be very low (39.5%) to low (20.7%) and had a neutral attitude about the necessity of hospitalization when being infected with DF (60.9%). A total of 17.6%, 9.8% and 6.6% of respondents reported frequently changing water, properly disposing of waste and covering water storage containers to eliminate larvae. Gender, education level, duration of illness and travel history were correlated with knowledge. Occupation, the presence of DF in the neighborhood, mosquito density at home and DF symptom severity were associated with attitudes. Occupation, mosquito density at home, type of patient, knowledge and attitudes were associated with practices. To enhance the KAP towards DF, further efforts should first be directed to improve knowledge through education, especially at the school level and people in less developed areas.

## 1. Introduction

According to the World Health Organization (WHO), among arboviral infection diseases, dengue is considered to be one of the most common types in the world; with approximately 390 million infected cases yearly, and 40% of the world population living in at-risk areas [1,2]. In many parts of the tropics and subtropics, dengue is endemic especially during rainfall season which is the breeding season of the *Aedes* mosquito [3]. Incidence dramatically increased to 2 million cases from 2008 to 2015 among the American, South-East Asian and Western Pacific regions, and extended to some European countries due to globalization and traveling [1]. Exhibiting symptoms similar to the flu, dengue fever (DF) can progress to severe and life-threatening stages which involves severe bleeding, respiratory and organ impairment [4]. Along with morbidity and fatality, DF poses a significant economic burden on the individuals and countries infected, partly since the disease dominantly affects young adults, those are likely to be working and ‘bread-winners’ in the family [5,6,7].

The knowledge, attitudes and practices (KAP) of the general population are the most critical factors on preventing the infection of dengue virus [8,9]. A study in Malaysia found that lack of knowledge on the dengue transmission and its preventive methods can increase the chance of spreading DF [10]. Moreover, the study of Rozita et al. indicated the need to expand DF preventive knowledge to control dengue outbreaks [11]. Besides, it is also essential to investigate DF patients’ health service seeking behavior [12]. Effective treatment of dengue fever has largely depended on appropriate and early medical care by experienced clinicians to avoid complications and reduce the fatality rate [4]. Early diagnosis and treatment can reduce the fatality rate of DF patients from 20% to 1% or less [13].

Located in the subtropical climate and undergoing unplanned rapid urbanization, Vietnam has experienced a number of dengue transmission outbreaks in recent years [14]. Specifically, the number of DF cases increased in 2017. In the first seven months of 2017, there were over 58,000 cases of DF, including more than 49,000 hospitalizations and 17 deaths, which showed an increase of 11.2% as compared to the same period in 2016 [15]. The number of DF cases was extremely high during rainfall season in Northern Vietnam. According to the Vietnam National Institute of Hygiene and Epidemiology, in 2017, there were 59,063 DF cases in Northern Vietnam, approximately eight times higher than in 2016 with 7289 DF cases [16]. Immediate efforts in strengthening prevention capacity have been made by the Vietnam Ministry of Health, including instructions to improve public awareness of and response to the disease [17]. Though such public campaigns have yielded positive initial results [17], longer-term prevention initiatives would require further study to understand the KAP of the public and DF prevention in Vietnam. Our study aimed to explore this under-researched topic, with a focus on KAP of DF patients, while also investigating the potential associated factors of KAP.

## 2. Materials and Methods

### 2.1. Study Design and Sampling Method

From September to November 2017, a cross-sectional study was conducted to assess KAP and associated factors related to DF among patients who had fever symptoms in the Infectious Diseases Department (IDD) of Bach Mai Hospital. Bach Mai hospital is the leading general hospital in Northern Vietnam, serving a diverse pool of patients, many coming from health facilities in regional or communal areas, being referred or transferred due to the severity of their conditions [18]. Since the beginning of July 2017, the IDD received an average of 50 to 70 DF patients daily, of which 1/3 required hospitalization [19]. In August 2017 the department received approximately 1189 cases [19].

### 2.2. Participants Recruitment

The participants were included in the study if they met the following eligibility criteria: (1) Patients visited IDD; (2) had DF or DF symptoms; (3) agreed to participate in this interview by giving written consent; (4) were able to answer the questionnaire in 10–15 minutes.

### 2.3. Data Collection

We used a convenience sampling method. The eligible patients were introduced to the research and gave written informed consent prior if they agreed to enroll. Face-to-face interviews were employed using a structured questionnaire and included questions about epidemiological characteristic, knowledge, attitude, practices regarding DF and DF prevention. All the researchers were working in the medical field and had been trained by experts to ensure the quality of data. Each interview lasted between 10–15 minutes. To ensure the confidentiality of the interview, we invited patients to a private area of the clinic. A total of 330 patients agreed to participate in the study.

### 2.4. Measurements and Instruments

We interviewed the patients to collect the following information:

#### 2.4.1. Social-Demographic Characteristics

The collected demographic data included age, gender, education, marital status, employment, living area.

#### 2.4.2. Clinical and Epidemiological Characteristics

We collected clinical data of participants including whether they were an in-patient or out-patient, the level of severity, the coexistence of other comorbidities and the duration of DF. The epidemiological characteristics were also collected. The information collected included whether there was DF cases in the patients’ neighborhood and had any relatives with DF. We collected data about their trip to other local areas in last 15 days. The patients also reported mosquito density at home from very low to very high level.

#### 2.4.3. Knowledge, Attitude, Practices Regarding Dengue Fever

● Knowledge about DF

We investigated participants’ knowledge about DF by asking if participants could identify clinical symptoms; the means of transmission; the basic therapies to prevent mosquitoes and larvae. Each correct answer regarding clinical symptoms, the DF transmission vector (mosquitoes), and methods to kill larvae and prevent mosquitoes counted as 1 point. The knowledge score ranged from 0–19 points.

● Attitude regarding DF

We asked the respondent’s feeling about the severe levels of DF disease, the necessity of hospitalization due to DF. Each question used a Likert scale with five levels. The scores for each item ranged from 0 (lowest) to 5 (highest). The attitude toward dengue fever score ranged from 0–15 points.

● DF Prevention Practices

We gathered information about how they controlled mosquitoes and larvae in their house, and whether a patient with DF symptoms went to a medical facility immediately. Each action that patients performed to kill larvae and prevent mosquitoes counted as 1 point. The DF prevention practices score ranged from 0–11 points.

### 2.5. Statistical Analysis

Data analysis was performed using Stata version 12.0 (Stata Corp. LP College Station United States of America). Categorical variables were interpreted using frequency and percentage. The continuous categories were interpreted using mean and standard deviation (SD). A Tobit regression model with a threshold of 0.2 was applied to determine factors associated with the knowledge, attitude and practice score of DF and DF prevention. A *p*-value equal to 0.05 or less was considered statistically significant.

### 2.6. Ethical Approval

The protocol of this study was reviewed and approved by the Institutional Review Board of Bach Mai Hospital.

## 3. Results

Table 1 describes the sociodemographic characteristics of respondents. The average age of the population was 31.6 years old (±12.7). Out of 330 participants, most lived in the urban area (77.9%) and attained above high school education (51.5%). A total of 25.7% of participants were freelancers, 23% were students, and 19.7% were white-collar workers.

Table 2 illustrates the clinical and epidemiological characteristics of the respondents at IDD at Bach Mai Hospital, Vietnam. More than half of respondents were not required to stay overnight at the hospital (55.3%) and reported being clinically symptomatic (68.5%), The average duration of DF was 5.5 days (±4.3). One-third of the respondents reported having comorbidities (34.2%). There were 43.1% of respondent reported having DF in their neighborhoods and 19% had relatives with DF. The majority of respondents reported very low to low density of mosquitoes at home (61%). Meanwhile, 14.3% of respondents reported traveling to other places in the past 15 days; whereas, 6.8% had relatives going to other places in the past 15 days.

Dengue-related knowledge is shown in Table 3. On the scale of 0–19 points, the average knowledge score was 4.6 (±2.1). The majority of respondents identified initial symptoms of DF as having a fever for more than two days (85.5%) and having a headache and joint pains (55.5%). The majority of respondents knew that being bitten by mosquitoes caused DF (90.5%). In terms of vector control, one-third of respondents knew that larvae could be killed by frequently changing water (37.7%). Other answers such as disposing of waste properly, using fish, tightly covering water storage containers and adding salt to water were reported by 18.6%, 10.4%, 9.8% and 2.2% of respondents respectively. To prevent mosquitoes, the majority of respondents knew insect repellent spray (63%) and mosquito net (35.4%) were prevention methods. Other methods were identified such as using mosquito repellent coils, house sanitation, environmental sanitation, clearing bushes and applying insect repellent body lotion.

Regarding attitude toward DF, the majority of respondents believed that DF was dangerous to very dangerous (91.1%). A high proportion of them perceived very low to low risk of DF infection (60.2%). Additionally, more than half of the patients had a neutral attitude about the necessity of hospitalization when being infected with DF (60.9%). The mean attitude score was 9.2 (±2.4) (Table 4).

Table 5 presents practices toward DF among patients at Bach Mai Hospital, Vietnam. In terms of larvae control at home (domestically), the proportion of correspondents reporting frequently changing water, properly disposing of waste and covering water storage containers were 17.6%, 9.8%, and 6.6%, respectively. In term of mosquitoes control and prevention, using a mosquito net and insect repellent spray were the most highly reported by respondents (49.2% and 48.3% respectively). The practice score among patients with DF was 1.5 (±1.4). Besides vector control and prevention methods, the majority of respondents also reported they would go to health facilities if they developed DF symptoms (61.6%). Moreover, 17.1% of respondent took drug/antibiotic without going to health facilities.

Table 6 presents the factors associated with KAP regarding DF. Compared to those with an education level less than high school, high school graduates were more likely to have better knowledge regarding DF (Coef = 1.28; 95% CI = 0.53–2.04). Those who were male, being sick longer, and traveled to other places in the past 15 days were more knowledgeable about DF than their counterparts. Being a farmer or blue-collared worker was associated with a poorer attitude toward DF compared to being unemployed (Coef= −1.15; 95% CI= −2.01–0.28). The density of mosquitoes at home and unawareness of DF cases present in the neighborhoods were negatively correlated with the attitude score. Those who experienced symptomatic DF were more likely to have a better attitude toward the disease (Coef = 0.86; 95% CI = 0.15–0.57). Compared to the unemployed population, freelancers were more likely to have good prevention practices (Coef = 0.53; 95% CI = 0.14–0.92). Similarly, the density of mosquitoes was also positively associated with prevention practices (Coef = 0.78; 95% CI = 0.03–0.68). People who had better knowledge were more likely to have a more serious attitude toward DF (Coef = 0.29; 95% CI = 0.16–0.42) and better prevention practices (Coef = 0.44; 95% CI = 0.35–0.53).

## 4. Discussion

Our study found that the majority of participants possess basic knowledge about DF, consider the illness to be serious and practice some level of prevention and treatment seeking. There has been, however, a lack of knowledge and practices regarding measures to eliminate larvae and mosquitos in living spaces. The higher knowledge score was found to be associated with the level of education, while reported mosquito density at home was found to negatively correlate with attitudes and positively correlate with practices.

The majority of participants correctly identified main initial DF symptoms (having more than two days fever and headache/joint pains) as well as the cause of illness, suggesting general public awareness of DF. This result is higher than that found in previous research conducted in Tan Trieu Commune and Dai Ang Commune, Thanh Tri District of Hanoi in 2016 [20]. Such level of basic knowledge may be a result of consistent efforts conducted by the health authority; the Ministry of Health has distributed and enforced detailed guidelines of DF prevention annually, especially in areas where the disease has been the most severe [17]. Nonetheless, knowledge of the measures to kill larvae and mosquitoes is still limited. Most respondents were unaware that keeping a high level of sanitation (proper waste disposal, house sanitation, etc.) and limiting open, dirty water surfaces in living spaces can be effective prevention methods. This problem posed a challenge in dengue prevention especially with tropical characteristics of weather in Hanoi, with a humid and wet rainy season that would be in favor of spreading dengue [14].

Regarding attitudes towards DF, two-thirds of participants reported considering it a severe disease, however a large proportion perceived their risk of having dengue fever to be very low to low while most believed that hospitalization was neither necessary nor unnecessary. Similar findings were found in cross-sectional studies conducted in Yemen and Thailand [8,21]. Such an attitude may hinder the efforts in dengue prevention, as preventive measures may be deemed not necessary by healthy people who did not consider themselves to be at risk of dengue.

Although adequate measures to kill mosquitoes at home have been reportedly used among our participants, the proportion of respondents taking measures to kill larvae at home was rather low, with the most popular method being the frequent changinging of water conducted by 17.6% of participants. Limited practices in preventing mosquito breeding have reflected the gap in knowledge also discovered in this study. Thus, to improve the practices of dengue prevention that would support limiting the spreading of the disease in the community, more efforts should first be given to enhancing the knowledge of the public, especially regarding measures of prevention. This suggestion is further supported by the finding of this study which showed a positive correlation between knowledge score and attitude and practices scores. Many studies have also provided evidence showing that the more people know about DF, the better attitudes and practices they have toward the disease [22,23].

Our results further aided the argument that education is positively associated with better disease prevention and control, which has been highlighted by an extensive number of studies. Specifically, current literature states that school-based health education for dengue control provides good knowledge of DF signs and symptom as well when and how to seek treatment [24,25,26]. Meanwhile, occupation was found not to be associated with knowledge, but attitude and practices regarding DF. Farmers and workers were more likely to have a poor attitude regarding DF. Even though general literacy has been improved significantly in Vietnam, a large proportion of farmers and workers who come from rural and ethnic minorities remained undereducated [27]. Those who had blue-collared jobs often had a lack of education and health literacy. Adequate health literacy allows individuals to understand and make a decision in health care, disease prevention and healthy behaviors [28]. Similarly, Hairi et al. found an association between literacy level and DF related knowledge and practice among Malaysian rural populations [29].

In this study, we found that people who reported low density of mosquitoes at home have a poorer attitude regarding DF as compared to people who reported the very minimal number of mosquitoes. This can partially be explained by the fact that living in areas with high-density of mosquitos may induce familiarity of people with mosquitoes and mosquito bites, possibly leading to them perceiving such bites as normal [30]. Such an attitude was found to be rather common in groups with lower socio-economic status [30]. In addition, people who reported a high density of mosquitoes were found more likely to practice prevention methods. Similar to the previous study, this can be explained by the fact that people who live in high-risk or endemic areas have more serious perceptions of mosquito-borne diseases such as DF, malaria and West Nile virus [31].

Several implications can be drawn from the findings of this study. First, education on disease and disease prevention at the school level should receive attention and support from the health authority, especially in rural or less developed areas where schools play a primary role in providing knowledge and information. In addition, when designing and implementing public mass campaigns and posters regarding DF, the level of literacy and cognitive understanding of the concerned population should be taken into account and incorporated in the final product. The use of media, especially social media, should also be considered, however with care, such that information can be more far-reaching without being misleading for targeting an incorrect group of the population.

Several limitations need to be noticed when interpreting the findings. First, the study used a convenient sampling method to recruit participants at the Bach Mai Hospital. Thus, this may limit the generalizability of the study. Second, socially desirable bias should also be considered as participants answered questions. To minimize the bias, interviewers were well trained to recognize the bias. Third, the reported KAP score might be higher than the general population because the study took place in the urban area where people might have more access to DF-related information. Moreover, the survey was employed on DF patients. There are possibilities that these patients searched for DF information. Last but not least, this is a cross-sectional study; therefore, we could not conclude causality of the relationship between KAP and associated factors.

## 5. Conclusions

This study discovered that, although the participants had a basic knowledge regarding dengue transmission vector and symptoms, and were generally aware of the seriousness of the disease and practiced some level of prevention, much remains to be done to enhance the capacity of the community to combat dengue and dengue fever. The gap in prevention knowledge, attitude towards the risk of being infected, and practices of eliminating sources of mosquitoes, to name a few, can be addressed through further education efforts. The literacy level and understanding of the targeted population, especially in less developed areas, should be considered when designing and implementing education campaigns and programs to ensure effectiveness.

## Figures and Tables

**Table 1:** Sociodemographic characteristics of respondents (*n* = 328).

Characteristic	*n*	%
**Total**	330	100.0
**Education**		
Under high school	64	20.3
High school	89	28.2
Above high school	163	51.5
**Marital Status**		
Single	154	47.2
Married	172	52.8
**Employment**		
Unemployment	22	6.7
Freelance	85	25.7
White-collars	65	19.7
Farmer, worker	34	10.3
Student	76	23.0
Others	48	14.6
**Living area**		
Urban	257	77.9
Rural	73	22.1
	**Mean**	**SD**
Age	31.6	12.7

**Table 2:** Clinical and epidemiological characteristics.

Characteristic	*n*	%
**Types of patients**		
In-patient	143	44.7
Out-patient	177	55.3
**Level of severity**		
Mild	52	15.8
Symptomatic	226	68.5
Severe	52	15.8
Having comorbidities	113	34.2
Having DF in the neighborhood	140	43.1
Having relatives with DF	61	19.0
Traveling to other locals in the last 15 days	44	14.3
Having relatives traveling to other locals in the last 15 days	21	6.8
Mosquitoes density at home		
Very low	111	38.0
Low	68	23.3
Normal	40	13.7
High	59	20.2
Very high	14	4.8
	**Mean**	**SD**
**Duration of illness (days)**	5.5	4.3

**Table 3:** Knowledge about DF.

Characteristic	*n*	%
**Initial symptoms when infecting dengue fever**		
Having a fever for more than 2 days	272	85.5
Having a skin rash	63	19.8
Nose bleeding, bloody diarrhea, blood vomiting	29	9.1
Cold extremities	15	4.7
Stomachache	19	6.0
Restlessness	24	7.6
A headache, joint pains	176	55.5
**Causes of dengue fever**		
Bites of mosquitoes	287	90.5
Blood transfusion	2	0.6
Airborne	2	0.6
Foodborne	0	0.0
**Measures to kill larvae**		
Frequently changing water	120	37.7
Proper disposal of waste	59	18.6
Tightly covering water storage containers	31	9.8
Fish	33	10.4
Adding salt to the water	7	2.2
**Measures to prevent mosquitoes**		
Using mosquito repellent coils	20	6.3
Using mosquito net	113	35.4
Using insect repellent spray	201	63.0
House sanitation	25	7.8
Environmental sanitation, clearing bushes	20	6.3
Applying insect repellent body lotion	16	5.0
	**Mean**	**SD**
**Knowledge score (0–19 points)**	4.6	2.1

**Table 4:** Attitude toward DF.

Characteristic	*n*	%
**Level of the danger of dengue fever**		
Very dangerous	116	36.7
Dangerous	172	54.4
Neutral	23	7.3
Not dangerous	5	1.6
Completely not dangerous	0	0.0
**Self-perceived risk of dengue fever**		
Very high risk	9	2.8
High risk	40	12.5
Having risk	78	24.5
Low risk	66	20.7
Very low risk	126	39.5
**The necessity of hospitalization if suffering dengue fever**		
Very necessary	18	5.9
Necessary	64	21.2
Neutral	187	60.9
Not necessary	37	12.0
Completely not necessary	0	0.0
	**Mean**	**SD**
Attitude score (0–15 points)	9.2	2.4

**Table 5:** Practice toward DF.

Characteristic	*n*	%
**Measures to kill larvae at home**		
Frequently changing water	56	17.6
Proper disposal of waste	31	9.8
Tightly covering water storage containers	21	6.6
Fish	10	3.1
Adding salt to the water	1	0.3
**Measures to prevent mosquitoes at home**		
Using mosquito repellent coils	9	2.8
Using mosquito net	157	49.2
Using insect repellent spray	154	48.3
House sanitation	19	6.0
Environmental sanitation, clearing bushes	10	3.1
Applying insect repellent body lotion	27	8.5
**Solutions when having dengue fever symptoms**		
Go to health facilities	191	61.6
Track and go to health facilities if the health condition is more severe	49	15.8
Taking drug/antibiotic	53	17.1
Other	15	4.8
Do nothing	2	0.7
	**Mean**	**SD**
Practice score (0–11 points)	1.5	1.4

**Table 6:** Associated factors with KAP about DF.

Characteristic	Knowledge Score		Attitude Score		Practice Score	
	Coef.	95%CI	Coef.	95%CI	Coef.	95%CI
**Gender (Male vs. Female)**	0.55 **	0.05; 1.05				
**Education (vs. less than high school)**						
High school	1.28 ***	0.53; 2.04				
>High school	0.89 **	0.21; 1.57	0.35	−0.18; 0.88		
**Marital status (Having spouse/partner vs. Single)**	0.46 *	−0.06; 0.97				
**Occupation (vs. Unemployed)**						
Freelancer					0.53 ***	0.14; 0.92
White-collars					0.36	−0.07; 0.79
Farmer, worker	−0.64	−1.47; 0.19	−1.15 ***	−2.01; −0.28		
**Duration of illness (days)**	0.08 **	0.00; 0.15	0.06	−0.02; 0.14		
**Having DF in neighborhood (vs. Yes)**						
No					−0.30 *	−0.64; 0.04
Unknown			−0.97 **	−1.71; −0.23		
**Level of severity (vs Mild)**						
Symptomatic			0.86 **	0.15; 1.57		
**Traveling to other locals in the last 15 days (Yes vs. No)**	0.70 **	0.02; 1.38	−0.69 *	−1.43; 0.04	−0.41	−0.92; 0.11
**Having relatives traveling to other locals in the last 15 days (Yes vs. No)**					−0.55	−1.30; 0.20
**Mosquitoes density at home (vs. Very low)**						
Low			−0.65 **	−1.26; −0.04		
Very high					0.78 **	0.03; 1.53
**Types of patients (Outpatient vs. Inpatient)**					0.34 **	0.01; 0.68
**Knowledge score**			0.29 ***	0.16; 0.42	0.44 ***	0.35; 0.53

*** *p* < 0.01, ** *p* < 0.05, * *p* < 0.1.
